# *PLCE1* Polymorphisms Are Associated With Gastric Cancer Risk: The Changes in Protein Spatial Structure May Play a Potential Role

**DOI:** 10.3389/fgene.2021.714915

**Published:** 2021-08-31

**Authors:** Xi’e Hu, Jintong Jia, Zhenyu Yang, Songhao Chen, Jingyi Xue, Sensen Duan, Ping Yang, Shujia Peng, Lin Yang, Lijuan Yuan, Guoqiang Bao

**Affiliations:** ^1^Department of General Surgery, The Second Affiliated Hospital of Air Force Medical University, Xi’an, China; ^2^Singleron Biotechnologies, Nanjing, China; ^3^The Second Clinical Medical College, Shaanxi University of Chinese Medicine, Xianyang, China

**Keywords:** gastric cancer, *PLCE1*, polymorphism, risk, prognosis, bioinformatics, protein structure

## Abstract

**Background:**

Gastric cancer (GC) is one of the most significant health problems worldwide. Some studies have reported associations between Phospholipase C epsilon 1 (*PLCE1*) single-nucleotide polymorphisms (SNPs) and GC susceptibility, but its relationship with GC prognosis lacked exploration, and the specific mechanisms were not elaborated fully yet. This study aimed to further explore the possible mechanism of the association between *PLCE1* polymorphisms and GC.

**Materials and Methods:**

A case-control study, including 588 GC patients and 703 healthy controls among the Chinese Han population, was performed to investigate the association between SNPs of *PLCE1* and GC risk by logistic regression in multiple genetic models. The prognostic value of *PLCE1* in GC was evaluated by the Kaplan-Meier plotter. To explored the potential functions of *PLCE1*, various bioinformatics analyses were conducted. Furthermore, we also constructed the spatial structure of PLCE1 protein using the homology modeling method to analyze its mutations.

**Results:**

Rs3765524 C > T, rs2274223 A > G and rs3781264 T > C in *PLCE1* were associated with the increased risk of GC. The overall survival and progression-free survival of patients with high expression of PLCE1 were significantly lower than those with low expression [HR (95% CI) = 1.38 (1.1–1.63), *P* < 0.01; HR (95% CI) = 1.4 (1.07–1.84), *P* = 0.01]. Bioinformatic analysis revealed that *PLCE1* was associated with protein phosphorylation and played a crucial role in the calcium signal pathway. Two important functional domains, catalytic binding pocket and calcium ion binding pocket, were found by homology modeling of PLCE1 protein; rs3765524 polymorphism could change the efficiency of the former, and rs2274223 polymorphism affected the activity of the latter, which may together play a potentially significant role in the tumorigenesis and prognosis of GC.

**Conclusion:**

Patients with high expression of *PLCE1* had a poor prognosis in GC, and SNPs in *PLCE1* were associated with GC risk, which might be related to the changes in spatial structure of the protein, especially the variation of the efficiency of PLCE1 in the calcium signal pathway.

## Introduction

Gastric cancer (GC) is becoming a worldwide problem year by year, endangering human life and health severely. It was estimated that over one million new GC cases occurred in 2018 and about 783 000 patients died of that, making GC the fifth most frequently diagnosed cancer and the third deadliest cancer worldwide (Bray et al., 2018). China has a large number of GC patients, with a 5-year overall survival (OS) of less than 25% (Chen et al., 2016; Zeng et al., 2018). The pathogenesis of GC is still unclear till now, but some risk factors have been reported, such as helicobacter pylori (Shimizu et al., 2014; Plummer et al., 2015; Jukic et al., 2021), Epstein-Barr virus infection (Camargo et al., 2014), low consumption of vegetables and fruits, high intake of salts and pickles, smoking and obesity (Lunet et al., 2007; Lin et al., 2014; Li et al., 2019). However, these research results are far from enough for us to understand the oncogenesis and susceptibility mechanism of GC.

In recent years, the genomic analysis of gastric tumors has highlighted the importance of its gene heterogeneity; and differentiations of GC molecular subtypes may be the key to guiding early diagnosis strategies, identifying new therapeutic targets, and predicting the prognosis of patients. In the last decade, single nucleotide polymorphism (SNP) analysis has been extensively used to screen candidate gene and detect various complex human diseases, providing a way to identify genetic loci associated with the heterogeneity of cancers.

Phospholipase C epsilon 1 (*PLCE1*) gene is one of the large-scale candidate genes located at 10q23 and served as a member of the human phosphoinositide-specific phospholipase C family (Song et al., 2001), which exerts an enormous function on growth, differentiation, and oncogenesis (Citro et al., 2007; Bunney and Katan, 2010; Gresset et al., 2012). The most-reported SNPs in *PLCE1* were rs2274223 and rs3765524, which have a significant value in increasing the risk of gastrointestinal tumor progression (Cui et al., 2014a; Mocellin et al., 2015; Mou et al., 2015; Xue et al., 2015; He et al., 2016; Gu et al., 2018). However, relevant studies of the associations between *PLCE1* and GC susceptibility remain inconsistent presently, and the prognostic value of *PLCE1* in GC is unclear; moreover, the specific mechanism between SNPs and GC risk is elusive now. Thus, further studies are still necessary.

This study aimed to analyze the relationship between three SNPs (rs3765524, rs2274223, and rs378126) in the *PLCE1* gene and GC susceptibility by a case-control study in the Chinese Han population firstly; then we explored the prognostic value of *PLCE1* in GC using online databases; finally, we tried to explain the correlation mechanism between the SNPs in *PLCE1* and the risk and prognosis of GC from the perspective of variable bioinformatics and protein spatial structure changes. We hope to make a contribute to the further exploration on the possible mechanism of the association between *PLCE1* polymorphisms and GC.

## Materials and Methods

### Study Population

A case-control study was conducted, including 588 patients with GC (392 males and 196 females) and 703 healthy control subjects (396 males and 307 females). All subjects were genetically related to Chinese Han. Patients with histologically confirmed GC in the Second Affiliated Hospital of Air Force Medical University from January 2015 to January 2019 were enrolled. The exclusion criteria for patients were: Patients who had a family history (three generations) of tumors; Those who had received radiotherapy or chemotherapy before blood sampling collection; Patients with any other digestive diseases or caused by metastasis of other cancer. Additionally, the healthy controls were randomly recruited from the physical examination center of the same hospital during the same period when they visited for an annual health examination. When recruiting healthy participants, we investigated the demographic information by personally interviewing through a structured questionnaire by trained personnel, including age, gender, residential region, ethnicity, and family history of cancer and other diseases. The healthy participants who had a family history of cancer were also excluded from the study. After that, we collected 5 mL peripheral blood of each subject to detect the SNPs of the *PLCE1* gene for our research. All participants were voluntarily recruited and provided written informed consent before taking part in this study. All research analyses were performed following the approved guidelines and regulations. This study was approved by the Research Ethics Committee of the Second Affiliated Hospital of Air Force Medical University (K201501-05) and abided by the Declaration of Helsinki.

### Genotyping

Agena MassARRAYAssay Design 4.0 software was used to design the multiplexed SNP Mass EXTEND assay. The *PLCE1* gene rs3765524, rs2274223, and rs3781264 polymorphisms were genotyped on the Agena MassARRAY RS1000 platform according to the standard protocol (Applied Biosystems, Foster City, CA, United States). Then, Agena Typer 4.0 software was applied to analyze and manage our data.

### Bioinformatics Analysis

#### The Prognostic Value of *PLCE1* in GC

The Kaplan Meier (K-M) plotter^1^ was used to evaluate the prognostic value of mRNA expression of *PLCE1*in GC patients. They were divided into high- and low-expression groups according to median values of mRNA expression and validated by K-M survival curves, with the hazard ratio (HR) with 95% confidence intervals (CIs) and Logrank *P*-value.

#### *PLCE1* Associated Genes Screening and Enrichment Analysis

STRING database^2^ (Szklarczyk et al., 2019) was applied to detect co-expression genes with *PLCE1* in GC, and Cytoscape software (Smoot et al., 2011) was used to explore and construct protein-protein interaction (PPI) network. Gene ontology (GO) enrichment, including biological process (BP), cellular component (CC) and molecular function (MF), and Kyoto Encyclopedia of Genes and Genomes (KEGG), were carried out to annotate PLCE1 functions by the Database for Annotation, Visualization, and Integrated Discovery (DAVID)^3^ (Huang da et al., 2009).

### Protein Homology-Modeling and Vitalization

The amino acid (aa) sequence of PLCE1 protein was obtained through NCBI.^4^ We used SWISS-MODEL^5^ to perform PLCE1 protein homology-modeling from its primary sequence (Schwede et al., 2003; Waterhouse et al., 2018). The protein with the highest coverage of the primary sequences was selected as the most homologous protein. We download the files of the constructed protein spatial structures in SWISS-MODEL and then opened them in PyMOL version 2.4^6^ for protein visualization to pave the way for PLCE1 protein spatial structure analysis (Arroyuelo et al., 2016; Yuan et al., 2016).

### Statistical Analysis

SPSS 26 (IBM SPSS Statistics, RRID:SCR_019096) software was applied to analyze the general characteristics of GC patients and healthy control groups. Welch’s *t*-test and the Pearson Chi-square test were applied to analyze differences of the basic characteristics between the two groups. The Pearson Chi-square test was also used to assess deviation from Hardy-Weinberg equilibrium (HWE) to compare the observed and expected genotype frequencies among the control subjects. Allele and genotype frequencies were compared between GC patients and healthy controls using the Pearson Chi-squared test and Fisher’s exact test. To evaluate the associations between *PLCE1* SNPs and the risk of GC, we calculated odds ratios (ORs) and 95% confidence intervals (CIs) adjusted by gender and age. Three different genetic models were applied (the codominant model, the dominant model and the recessive model) using PLINK software (PLINK, version 2.0, RRID:SCR_001757). *p-*value < 0.05 was considered statistically significant in all statistical tests in this study.

## Results

### Demographic Characteristics

The primary characteristics of all subjects were shown in Table 1. A total of 1,291 participants, including 588 GC patients and 703 healthy controls, were enrolled in this study. The mean age was 58.12 ± 11.66 years in GC patients and 48.57 ± 9.43 years in healthy controls, which indicated that the patients were elder than the healthy participants (*P* < 0.001). Besides, the scale of males was larger than females in the GC group (male to female is 66.67–33.33%), while the difference between males and females in the control group was minor (male to female is 56.33–43.67%). The difference in the distributions between GC patients and healthy controls suggested that the ORs and *p*-values need to be adjusted according to age and gender in subsequent analysis. Additionally, most of the participants in the study had an adverse family cancer history (cases, 96.3%; controls, 98.0%). Moreover, nearly one-third (30.3%) of patients were at an early stage (the carcinoma was confined to the gastric mucosa and submucosa).

**TABLE 1:** Basic demographic characteristics of gastric cancer patients and healthy controls.

**Characteristics**	**Patients (*n* = 588) (%)**	**Controls (*n* = 703) (%)**	***P*-value**
Age (years), mean ± SD	58.12 ± 11.66	48.57 ± 9.43	<0.001*
**Gender**			<0.001*
Male	392 (66.7)	396 (56.3)	
Female	196 (33.3)	307 (43.7)	
Tumor size (cm)	3.72 ± 2.12		
**Location**			
Cardia	141 (24.0)		
Body	211 (35.9)		
Pylorus	20 (3.4)		
Antrum	216 (36.7)		
**Borrmann**			
I	77 (13.1)		
II	56 (9.5)		
III	253 (43.0)		
IV	126 (21.4)		
V	59 (10.0)		
Unknown	17 (2.9)		
**Differentiation**			
Well	350 (59.5)		
Poor	238 (40.5)		
**T stage**			
T1	178 (30.3)		
T2	219 (37.2)		
T3	171 (29.1)		
T4	20 (3.4)		
**N stage**			
N0	294 (50.0)		
N1	141 (24.0)		
N2	75 (12.8)		
N3	78 (13.3)		
**M stage**			
M0	557 (94.7)		
M1	31 (5.3)		

*P-values were calculated using Welch’s t-test/Pearson Chi-square test.*

SD, standard deviation.

*P < 0.05 indicates statistical significance, which was marked in bold.

### Genotyping Analysis

The detailed information of the three selected SNPs, including roles, MAF, and HWE *P*-values, were listed in Table 2. These SNPs were genotyped successfully in further analysis. MAF of all SNPs was greater than 5%, and the observed genotype frequencies of all SNPs in the control groups were in HWE (*P* > 0.05).

**TABLE 2:** Basic information of three SNPs in PLCE1 on 10q23.33.

**SNP ID**	**Position**	**Alleles (A/B)**	**Role**	**MAF**		**O (HET)**	**E (HET)**	***P*-value**
				**Case**	**Control**			
rs3765524	96,058,298	T/C	Coding exon	0.244	0.214	0.331	0.336	0.654
rs2274223	96,066,341	G/A	Coding exon	0.246	0.213	0.332	0.335	0.822
rs3781264	96,070,375	C/T	Intron	0.190	0.142	0.240	0.243	0.756

*Sites with HWE, P < 0.05, are excluded. P-values calculated with two-sided Pearson Chi-square test.*

*SNP, single nucleotide polymorphism; Alleles (A/B), Alleles (minor/major); MAF, minor allele frequency; O (HET), observed heterozygosity; E (HET), expected heterozygosity; HWE, Hardy-Weinberg equilibrium.*

*P < 0.05 indicates statistical significance.*

Differences in the frequency distribution of SNPs genotypes and alleles between GC patients and healthy controls were compared by Pearson Chi-squared test and odds ratios (ORs) to evaluate the associations with GC risk, as displayed in Supplementary Table 1. The minor allele of each SNP as a risk factor was compared to the wild-type (major) allele. Remarkably, we found that the allele frequency of rs2274223 locating in the exon region was significantly different between GC cases and healthy controls [OR (95% CI) = 1.20 (1.00–1.45), *P* = 0.048]. What’s more, the genotype of rs3781264 in the intron region was also significantly different between the two groups [OR (95% CI) = 1.43 (1.16, 1.76), *P* = 0.001].

Then, we analyzed the associations between SNPs with the risk of GC, which was displayed in Table 3. Three polymorphisms (rs3765524, rs2274223, rs3781264) in *PLCE1* increased the risk of GC, which were identified through the dominant model (rs3765524, TT-TC vs. CC, OR = 1.28, 95% CI = 1.03–1.60, *P* = 0.034; rs2274223, GG-GA vs. AA, OR = 1.30, 95% CI = 1.04–1.62, *P* = 0.025; rs3781264, CC-CT vs. TT, OR = 1.53, 95% CI = 1.21–1.95, *P* = 0.001), and codominant model [rs3781264, CC vs. CT, OR (95% CI) = 1.01 (0.49–2.08), CC vs. TT, OR (95% CI) = 1.54 (0.76–3.13), *P* = 0.002).

**TABLE 3:** The relationships of five SNPs with gastric cancer according to the stratification adjusted by gender and age.

**SNP ID**	**Model**	**Genotype**	**Case (%)**	**Control (%)**	**OR (95%CI)**	***P*-value**
rs3765524	Allele	T	287 (24.40)	300 (21.34)	1.00	0.067
		C	889 (75.60)	1,104 (78.66)	1.19 (0.99, 1.43)	
	Codominant	T/T	29 (4.93)	34 (4.84)	1.00	0.080
		T/C	229 (50.85)	232 (33.00)	0.86 (0.51–1.47)	
		C/C	330 (56.12)	436 (62.02)	1.62 (0.96–2.72)	
	Dominant	T/T-T/C	258 (43.88)	266 (37.84)	1.00	0.034*
		C/C	330 (56.12)	436 (62.02)	1.28 (1.03–1.60)	
	Recessive	TT	29 (4.93)	34 (4.84)	1.00	0.955
		T/C-C/C	559 (95.07)	668 (95.02)	1.02 (0.61–1.69)	
rs2274223	Allele	G	289 (24.57)	299 (21.27)	1.00	0.048*
		A	887 (75.43)	1,105 (78.73)	1.20 (1.00, 1.45)	
	Codominant	G/G	29 (4.93)	33 (4.69)	1.00	0.064
		G/A	231 (39.29)	233 (33.14)	0.89 (0.52–1.51)	
		A/A	328 (55.78)	436 (62.02)	1.17 (0.70–1.96)	
	Dominant	G/G-G/A	260 (44.22)	266 (37.84)	1.00	0.025*
		A/A	328 (55.78)	436 (62.02)	1.30 (1.04–1.62)	
	Recessive	G/G	29 (4.93)	33 (4.69)	1.00	0.950
		G/A-A/A	559 (95.07)	669 (95.16)	1.05 (0.63–1.75)	
rs3781264	Allele	C	224 (19.05)	199 (14.15)	1.00	0.001*
		T	952 (80.95)	1207 (85.85)	1.43 (1.16, 1.76)	
	Codominant	C/C	17 (2.89)	15 (2.13)	1.00	0.002*
		C/T	190 (32.31)	169 (24.04)	1.01 (0.49–2.08)	
		T/T	381 (64.80)	519 (73.83)	1.54 (0.76–3.13)	
	Dominant	C/C-C/T	207 (35.20)	184 (26.17)	1.00	0.001*
		T/T	381 (64.80)	519 (73.83)	1.53 (1.21–1.95)	
	Recessive	C/C	17 (2.89)	15 (2.13)	1	0.489
		C/T-T/T	571 (97.11)	688 (97.87)	1.37 (0.68–2.76)	

*ORs and P-values were adjusted by age and gender.*

OR, odds ratio; CI, confidence interval.

*P < 0.05 indicates statistical significance, which was marked in bold.

### Bioinformatics Analysis of *PLCE1*

#### The Prognostic Value of *PLCE1* in GC

**FIGURE 1:**
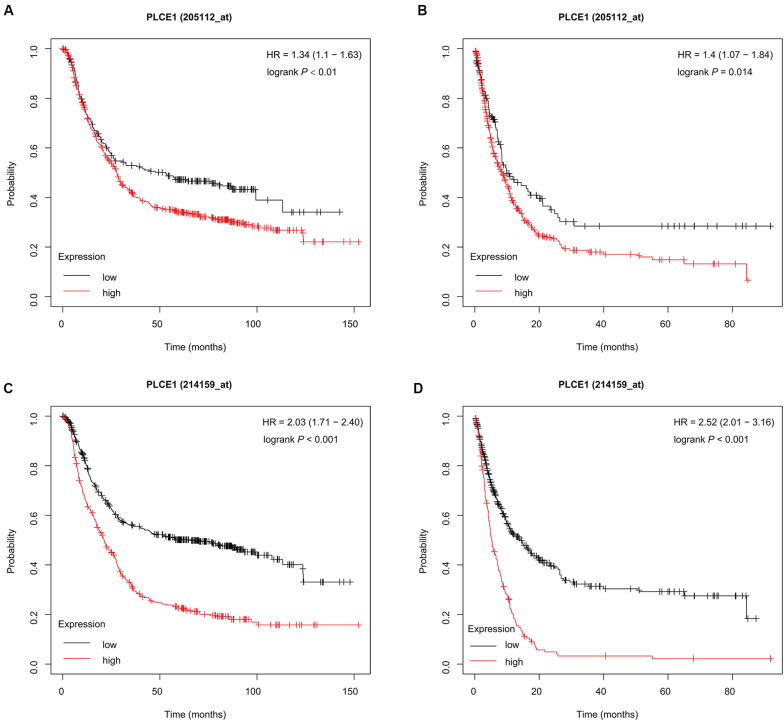
Prognostic characteristic (Kaplan-Meier plotter) of mRNA expression of PLCE1 in gastric cancer patients. The OS curves **(A,C)** and PFS curves **(B,D)** comparing patients with high-expression (red) and low-expression (black) of PLCE1 in gastric cancer by two probes (205112 and 214159) were plotted using the Kaplan-Meier plotter database according to the threshold of *P*-value of < 0.05.

The K-M plotter (Figure 1) showed that patients with *PLCE1* high-expression had lower OS and PFS in both two data sets [data set 205112, OS, HR (95% CI) = 1.34 (1.1–1.63), *P* < 0.01, PFS, HR (95% CI) = 1.4 (1.07–1.84), *P* = 0.01; data set 214159, OS, HR (95% CI) = 2.03 (1.71–2.40), *P* < 0.001, PFS, HR (95% CI) = 2.52 (2.01–3.16), *P* < 0.001], which indicated that *PLCE1* increased the risk of a poor prognosis in GC patients.

#### PLCE1 PPI Analysis

**FIGURE 2:**
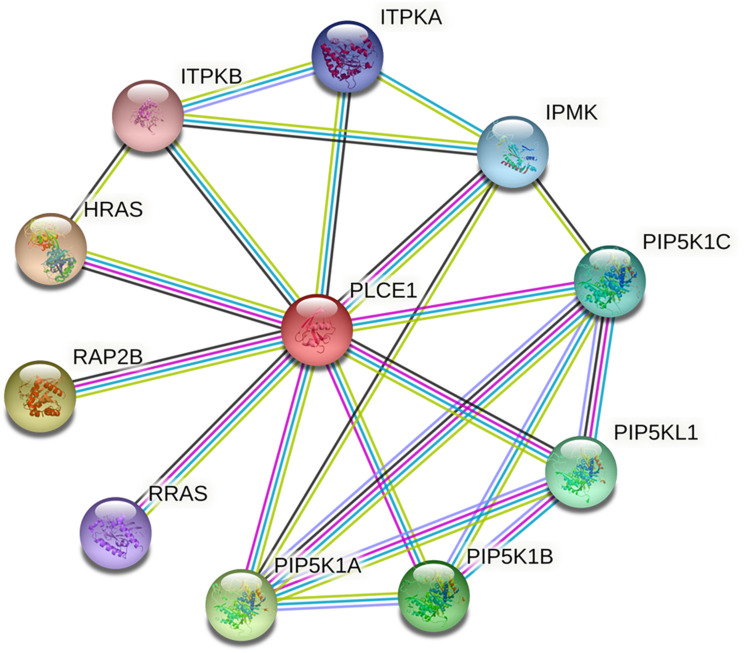
The core protein-protein interaction (PPI) network of PLCE1 constructed by STRING database. It was consisted by 11 nodes and 22 edges with the average node degree of 4 in gastric cancer. The nodes represent proteins; the edges represent interactions between proteins in the network. PLCE1, 1-phosphatidylinositol 4, 5-bisphosphate phosphodiesterase epsilon-1; HRAS, GTPase Hras; ITPKB, inositol-trisphosphate 3-kinase B; ITPKA, inositol-trisphosphate 3-kinase A; IPMK, inositol polyphosphate multikinase; PIP5K1C, phosphatidylinositol 4-phosphate 5-kinase type-1 gamma; PIP5KL1, phosphatidylinositol 4-phosphate 5-kinase-like protein 1; PIP5K1B, phosphatidylinositol 4-phosphate 5-kinase type-1 beta; PIP5K1A, phosphatidylinositol 4-phosphate 5-kinase type-1 alpha; RRAS, Ras-related protein R-Ras; RAP2B, Ras-related protein Rap-2b.

We investigated the PPI network of PLCE1 by STRING website, and we obtained the core network constructed by 11 nodes and 22 edges with an average node degree of 4 (*P* = 0.004; Figure 2). The interaction proteins with PLCE1 were PIP5K1A, PIP5K1B, PIP5K1C, PIP5KL1, IPMK, ITPKA, ITPKB, HRAS, RAP2B, and RRAS.

#### GO and KEGG Enrichment Analysis

**FIGURE 3:**
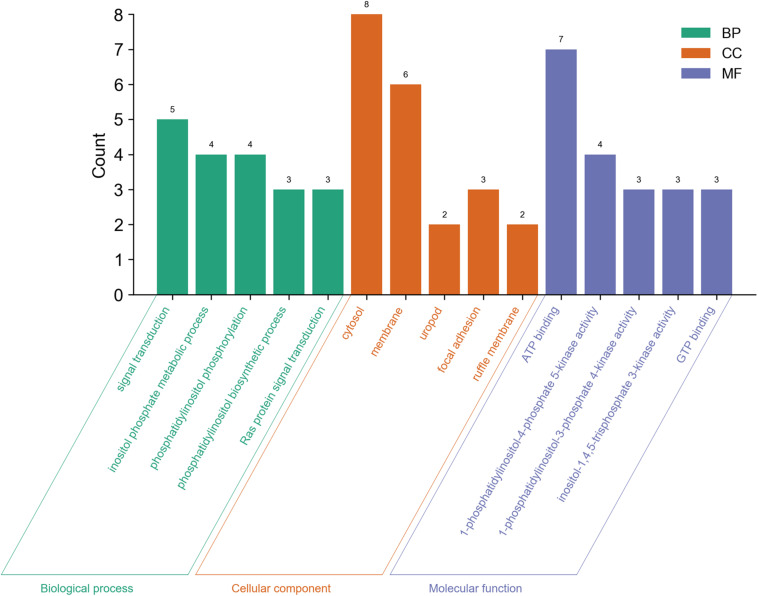
The GO enrichment analysis of PLCE1 and its co-expression genes by DAVID database. BP (biological process) was marked in green; CC (cellular component) was in orange; and MF (molecular function) was in purple.

**FIGURE 4:**
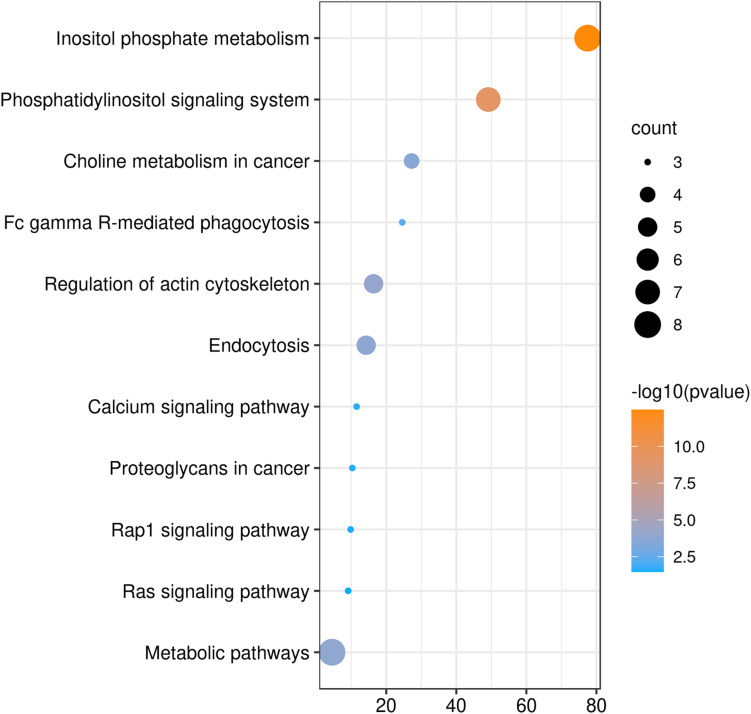
The KEGG enrichment analysis of PLCE1 and its co-expression genes by DAVID database. The size of the circle represents the counts of genes enriched, and the larger the circle, the more genes were enriched. From orange to blue, –10log (*P*-value) gradually decreased.

We also analyzed GO and KEGG enrichment by DAVID to explore the potential role of PLCE1. We found that the top five enrichments related to BP were signal transduction (GO:0007165), inositol phosphate metabolic process (GO:0043647), phosphatidylinositol phosphorylation (GO:0046854), phosphatidylinositol biosynthetic process (GO:0006661) and Ras protein signal transduction (GO:0007265) (Figure 3). Additionally, we found CC, such as cytosol (GO:0005829), membrane (GO:0016020), uropod (GO:0001931), focal adhesion (GO:0005925) and ruffle membrane (GO:0032587), were significantly associated with PLCE1. Moreover, PLCE1 also affected MF, including ATP binding (GO:0005524), 1-phosphatidylinositol-4-phosphate 5-kinase activity (GO:0016308), 1-phosphatidylinositol-3-phosphate 4-kinase activity (GO:0052811), inositol-1, 4, 5-trisphosphate 3-kinase activity (GO:0008440) and GTP binding (GO:0005525). KEGG enrichment analysis revealed that PLCE1 had a high correlation with inositol phosphate metabolism, phosphatidylinositol signaling system, choline metabolism in cancer, Fc gamma R-mediated phagocytosis, regulation of actin cytoskeleton, endocytosis, calcium signaling pathway, proteoglycans in cancer, rap1 signaling pathway and Ras signaling and metabolic pathways (Figure 4). All enrichment results could be attached in Supplementary Materials.

### PLCE1 Protein Spatial Structure Changes

**FIGURE 5:**
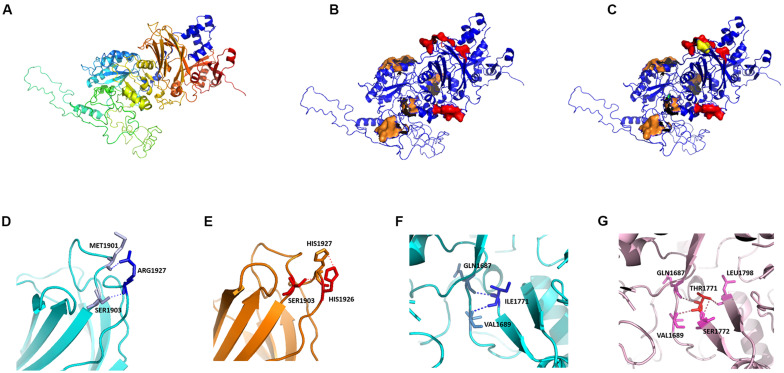
Protein homology modeling of PLCE1 by SWISS-MODEL. **(A)** The spatial protein model of the original (wild-type) PLCE1. From blue to red showed the coiled peptide chain from N- to C- terminal. **(B)** Exploration of crucial functional domains of PLCE1. The calcium ion binding pocket was marked in red, and the catalytic binding pocket was in orange. **(C)** The spatial protein model of the mutant PLCE1 protein. The mutation of rs2274223 (A > G) changed the 1927aa, which may affect the activity of the calcium-binding pocket (yellow). The mutation of rs3765524 (T > C) changed the 1771aa, which may influence the catalytic efficiency of the catalytic binding pocket (green). **(D)** The wild-type protein microenvironment analysis of PLCE1 on the single 1927aa site. ARG1927 formed two ionic bonds with MET1901 and SER1903, respectively, making the force between the two loops very tight. **(E)** The microenvironment analysis of the mutation (rs2274223 A > G) of PLCE1. Arg1927His-mutant of PLCE1 formed ionic bonds with these two aa residues; one of them was located on the loop of the 1927 site itself and formed a conjugate bond, making the attraction between the residues stronger than the wild-type. **(F)** The wild-type protein microenvironment analysis of PLCE1 on the single 1771aa site. Ile1771 formed two ionic bonds with Gln1687 and Val1689 residues, respectively. **(G)** The microenvironment analysis of the mutation (rs3765524 T > C) of PLCE1. Ile1771Thr mutant formed four ionic bonds with Gln1687, Val1689, Ser1772, and Leu1798, two of which located on the left loop and the others on the right loop, making the local structure more stable.

We modeled the primary PLCE1 protein by SWISS-MODEL. The original (wild-type) model of PLCE1 was shown in Figure 5A. The protein was colored from blue to red, representing the coiled peptide chain from N- to C-terminal. We found that the PLCE1 protein had two crucial functional domains, namely the calcium ion binding pocket (related to activity), which is composed of 1,873, 1,897, 1,926, 1,928, and 1,933 aa sites (red in Figure 5B), and the catalytic binding pocket (related to catalytic efficiency), consisting of 1,391, 1,392, 1,421, 1,423, 1,436, 1,470, 1,637, 1,639, 1,743, 1,770, and 1,772 aa sites (orange in Figure 5B).

Hence, the rs2274223 (A > G) changed the aa at the 1927 site, which may affect the activity of the calcium-binding pocket (yellow in Figure 5C). Similarly, the mutation of rs3765524 (T > C) enabled the aa at the 1,771 site to change, influencing the catalytic efficiency of the catalytic binding pocket (green in Figure 5C).

Interestingly, in further analysis of the impact of the single aa mutation on the protein microenvironment, we found that the ARG1927, in the wild type, formed two ionic bonds with MET1901 and SER1903, respectively (Figure 5D), making the interaction force between the two loops extremely tight. However, the mutation (A > G) of rs2274223 resulted in Arg1927His in PLCE1 protein, displayed in Figure 5E; although it still formed ionic bonds with these two aa residues after the mutation, one of them was located on the loop of the 1,927 site itself and formed a conjugate bond, causing the attraction between the residues to be stronger than the original one. Consequently, the loop in 1,927 would be tighter than before, and the calcium-binding pocket was more difficult to open after the mutation, leading to the decrease of the protein (PLCE1) activity.

Likewise, in the wild-type, Ile1771 formed two ionic bonds with Gln1687, Val1689 residues, respectively. The interaction force between the ionic bond and the left loop was tight, but no force existed between the loops on the right to “fix” (Figure 5F), so it would be easier for the dissociation in a solution or the local changes, facilitating the substrate entered the active center readily. However, rs3765524 (T > C) lead to the Ile1771Thr, which generated four ionic bonds with the four aa residues (Gln1687, Val1689, Ser1772, and Leu1798) in the surrounding space, two of which located on the left loop and the others on the right loop, making the local structure more stable, so the change of the catalytic pocket seemed to be more challenging (Figure 5G).

These variations mentioned above combined with the results of bioinformatics analysis indicated that SNPs in *PLCE1* could change the catalytic activity of the protein in Ca^2+^-related pathways, so more substrates (such as Ca^2+^) might be required to perform normal functions, which will be verified in our future studies.

## Discussion

As a common genetic variation in human genome, SNP is beneficial for understanding the possible relationships between tumors and individuals’ biological functions on a genomic scale. It provides a comprehensive tool for identifying candidate genes of cancer, offering fundamental knowledge for clinical diagnosis and revealing drug discovery for relevant genetic diseases; therefore, SNP is considered as a kind of commendable biological marker in diverse tumors (Engle et al., 2006).

Protein is an indispensable carrier of various biological activities and plays a crucial role in the smooth progress of diverse life courses. The primary structure of a protein is aa sequence, which is derived from gene transcription and translation. It is the basis of a high-order structure of a protein and determines the spatial structure and functional properties of a protein. When a SNP is present in a gene, the expressed aa sequence may change, resulting in a change in the spatial structure of the protein. Therefore, it is imperative to study the risk of SNPs and GC from the perspective of protein spatial structure changes, which will contribute to the research on the pathogenesis and prognosis of GC.

In this study, for the first time, we analyzed the correlation between SNPs and GC susceptibility and prognosis in terms of protein spatial structure changes. Firstly, we carried on a case-control study, and by detecting and analyzing the differences on SNPs of *PLCE1* between GC patients and healthy controls, we found that rs3765524 (C > T), rs2274223 (A > G), and rs3781264 (T > C) were related to the susceptibility of GC. Then, the K-M plotter demonstrated that high-expression of *PLCE1* was associated with poor survival in GC. To explore the potential function of PLCE1, we used a series of bioinformatics tools, investigating the PPI network, GO and KEGG of PLCE1, and found it played a potential role in the calcium signaling pathway. Furthermore, we constructed the primary and mutant protein spatial structures of PLCE1 by homology modeling method, and interestingly, we found that the changes of the protein spatial structure could reduce the catalytic activity, which might mainly influence its function in Ca^2+^-related pathways. Combined with the bioinformatic results of *PLCE1*, we speculated that *PLCE1* polymorphisms increase GC susceptibility by changing the spatial structure of PLCE1 protein, affecting its activity and catalytic efficiency in the calcium signaling pathway. This hypothesis will be verified in our future experiments.

As a member of the phospholipase C family of proteins, *PLCE1* encodes a phospholipase C enzyme which mediates the hydrolysis reaction of phosphatidylinositol-4,5-bisphosphate to produce the Ca^2+^-mobilizing second messenger inositol 1,4,5-triphosphate and the protein kinase C-activating second messenger diacylglycerol. It interacts with the proto-oncogene Ras among other proteins (Bunney et al., 2009). The expression of *PLCE1* was significantly related to tumor differentiation degree, invasion depth, lymph node metastasis and distant metastasis (Cui et al., 2014b; Cheng et al., 2017; Yu et al., 2020).

We confirmed the significance of the two SNPs previously reported, rs3765524 and rs2274223, and revealed another SNP in *PLCE1*, rs3781264, through genotyping and logistic regression in this case-control study was associated with the GC risk. Abnet et al. (2010) firstly used GWAS to identify those variants of *PLCE1* had a significant correlation with GC in the Chinese Han population Until now, an increasing number of studies have identified a shared susceptibility locus in *PLCE1* such as rs2274223 A > G and rs3765524 C > T for gastrointestinal cancer (Abnet et al., 2010; Umar et al., 2013; Cui et al., 2014b; Liu et al., 2014; Malik et al., 2014; Mocellin et al., 2015; He et al., 2016; Gu et al., 2018; Li et al., 2018; Liang et al., 2019; Xie et al., 2020), and the most reported SNP of *PLCE1* was the former, but the conclusions lack consistency. A meta-analysis showed that *PLCE1* rs2274223 polymorphism resulted in susceptibility to esophageal and GC in Asians (Umar et al., 2013). However, another study suggested that an increased association between rs2274223 and GC risk among Asian ethnic groups could only be observed in esophageal cancer rather than GC (Xue et al., 2015). The discrepancy probably results from considerable heterogeneity in these studies as well as gene-gene interaction and gene-environment interaction. A study (Liang et al., 2019) also confirmed our hypothesis at the protein level by immunohistochemistry (IHC), which confirmed that the PLCE1 protein expression was higher in group of rs3765524 CT/TT than in group of rs3765524 CC. Additionally, our study also showed that rs3781264, located on an intron region, had a potential relationship with GC risk, which was scarcely reported before. Hitherto, most the previous studies focus on the correlation between gene SNPs and cancer susceptibility or risk but never explore its mechanism further.

Currently, the diagnosis, treatment and prognosis of GC are usually based on a risk stratification system. The most efficient curative therapeutic option for GC patients is timely adequate surgical resection (Lutz et al., 2012). Besides, chemotherapy, as a way of second-line treatment, can improve overall survival (Kang et al., 2012). Although we have some understanding of carcinogenesis of GC, early diagnosis and appropriate therapy methods on GC patients still remain a major clinical challenge till now (Choi et al., 2003; Ang and Fock, 2014). It is essential for individuals to identify high-risk GC; thus, more precise gene loci associated with it should be explored. In this study, the K-M plotter analysis was performed in the online bioinformatics database, and both two probes showed that the patients with high mRNA expression of *PLCE1* would have a poorer prognosis. It was suggested that *PLCE1* might have the potential to be a biomarker for the prognosis of GC.

The function of a protein is significantly determined by the spatial structure, which is an indispensable part of protein research. In this study, we analyzed the changes of PLCE1 protein spatial structure after mutations by homology modeling method; and we found it had two important functional domains, calcium-binding pocket related to its protein activity and Ca^2+^ binding pockets associated with the efficiency of Ca^2+^, which were never reported before. Interestingly, the two SNP sites we focused on, rs2274223 and rs3765524, were located on these important domains. The mutation in rs2274223 affected the Ca^2+^ binding pockets, deregulating its bioactivity efficiency related to Ca^2+^, and the T > C change in rs3765524 resulted in the efficiency decrease in catalytic activity. All these above together altered the structure, stability, and function of PLCE1 protein. Therefore, by our research, we suppose that SNPs of *PLCE1* may have potential significance in the tumorigenesis and progression of GC, perhaps mainly attributed to the changes of the protein activity, but further studies are needed to confirm.

In summary, this study for the first time analyzes the correlation between SNPs of *PLCE1* and GC in terms of protein spatial structure changes, which has a great significance to the diagnosis and treatment for patients with GC. The more complex connections or the subtle crosstalk will be verified in our future paper, and actually, this experiment is being carried out in full swing.

There were some limitations in this study. Firstly, we selected only three SNPs of *PLCE1*, and more other potentially significant loci were not included in this case-control study. Secondly, the prognostic value of *PLCE1* was investigated in the patients from the online database but not the subjects included in our study, which probably caused background heterogeneity. Thirdly, the mechanism of potential significance in the tumorigenesis and progression of GC was based on the bioinformatic results and the protein homology modeling analysis but lack of experimental verification. Therefore, studies *in vitro* and *in vivo* are needed and will be performed in the future to confirm our results, and we hope to contribute to the era of precise diagnosis and individualized treatment of GC.

## Data Availability Statement

The raw data supporting the conclusions of this article will be made available by the authors, without undue reservation.

## Ethics Statement

The studies involving human participants were reviewed and approved by the Research Ethics Committee of The Second Affiliated Hospital of Air Force Medical University. The patients/participants provided their written informed consent to participate in this study.

## Author Contributions

XH, LYu, and GB designed the research. ZY, SC, JX, and SD performed the study. XH, JJ, SP, PY, LYu, and LYa analyzed the results. XH and JJ edited and commented on the manuscript. All authors contributed to the article and approved the submitted version.

## Conflict of Interest

The authors declare that the research was conducted in the absence of any commercial or financial relationships that could be construed as a potential conflict of interest.

## Publisher’s Note

All claims expressed in this article are solely those of the authors and do not necessarily represent those of their affiliated organizations, or those of the publisher, the editors and the reviewers. Any product that may be evaluated in this article, or claim that may be made by its manufacturer, is not guaranteed or endorsed by the publisher.

## Footnotes

1
http://kmplot.com/analysis/

2
https://www.string-db.org/

3
https://david.ncifcrf.gov/

4
https://www.ncbi.nlm.nih.gov

5
https://swissmodel.expasy.org

6
http://www.pymol.org/2/

## References

[B1] Abnet C. C. Freedman N. D. Hu N. Wang Z. Yu K. Shu X. O. (2010). A shared susceptibility locus in PLCE1 at 10q23 for gastric adenocarcinoma and esophageal squamous cell carcinoma. *Nat. Genet.* 42 764–767. 10.1038/ng.649 20729852 PMC2947317

[B2] Ang T. L. Fock K. M. (2014). Clinical epidemiology of gastric cancer. *Singapore Med. J.* 55 621–628. 10.11622/smedj.2014174 25630323 PMC4291998

[B3] Arroyuelo A. Vila J. A. Martin O. A. (2016). Azahar: a PyMOL plugin for construction, visualization and analysis of glycan molecules. *J. Comput. Aided Mol. Des.* 30 619–624. 10.1007/s10822-016-9944-x 27549814

[B4] Bray F. Ferlay J. Soerjomataram I. Siegel R. L. Torre L. A. Jemal A. (2018). Global cancer statistics 2018: GLOBOCAN estimates of incidence and mortality worldwide for 36 cancers in 185 countries. *CA Cancer J. Clin.* 68 394–424. 10.3322/caac.21492 30207593

[B5] Bunney T. D. Baxendale R. W. Katan M. (2009). Regulatory links between PLC enzymes and RAS superfamily GTPASES: signalling via PLCepsilon. *Adv. Enzyme Regul.* 49 54–58. 10.1016/j.advenzreg.2009.01.004 19534025

[B6] Bunney T. D. Katan M. (2010). Phosphoinositide signalling in cancer: beyond PI3K and PTEN. *Nat. Rev. Cancer* 10 342–352. 10.1038/nrc2842 20414202

[B7] Camargo M. C. Kim W. H. Chiaravalli A. M. Kim K. M. Corvalan A. H. Matsuo K. (2014). Improved survival of gastric cancer with tumour Epstein-Barr virus positivity: an international pooled analysis. *Gut* 63 236–243. 10.1136/gutjnl-2013-304531 23580779 PMC4384434

[B8] Chen W. Zheng R. Baade P. D. Zhang S. Zeng H. Bray F. (2016). Cancer statistics in China, 2015. *CA Cancer J. Clin.* 66 115–132. 10.3322/caac.21338 26808342

[B9] Cheng Y. Xing S. G. Jia W. D. Huang M. Bian N. N. (2017). Low PLCE1 levels are correlated with poor prognosis in hepatocellular carcinoma. *Onco Targets Ther.* 10 47–54. 10.2147/OTT.S126401 28031722 PMC5182043

[B10] Choi N. K. Youn K. E. Heo D. S. Lee S. M. Kim Y. Park B. J. (2003). Stomach cancer incidence, mortality and survival rate in Korean elderly pharmacoepidemiologic cohort (KEPEC) in 1994˜1998. *Cancer Res. Treat.* 35 383–390. 10.4143/crt.2003.35.5.383 26680963

[B11] Citro S. Malik S. Oestreich E. A. Radeff-Huang J. Kelley G. G. Smrcka A. V. (2007). Phospholipase CEPSILON is a nexus for Rho and Rap-mediated G protein-coupled receptor-induced astrocyte proliferation. *Proc. Natl. Acad. Sci. U.S.A.* 104 15543–15548. 10.1073/pnas.0702943104 17878312 PMC2000505

[B12] Cui X. B. Pang X. L. Li S. Jin J. Hu J. M. Yang L. (2014a). Elevated expression patterns and tight correlation of the PLCE1 and NF-κB signaling in Kazakh patients with esophageal carcinoma. *Med. Oncol.* 31:791. 10.1007/s12032-013-0791-5 24307345

[B13] Cui X. B. Peng H. Li S. Li T. T. Liu C. X. Zhang S. M. (2014b). Prognostic value of PLCE1 expression in upper gastrointestinal cancer: a systematic review and meta-analysis. *Asian Pac. J. Cancer Prev.* 15 9661–9666. 10.7314/apjcp.2014.15.22.9661 25520085

[B14] Engle L. J. Simpson C. L. Landers J. E. (2006). Using high-throughput SNP technologies to study cancer. *Oncogene* 25 1594–1601. 10.1038/sj.onc.1209368 16550159

[B15] Gresset A. Sondek J. Harden T. K. (2012). The phospholipase C isozymes and their regulation. *Subcell. Biochem.* 58 61–94. 10.1007/978-94-007-3012-0_3 22403074 PMC3638883

[B16] Gu D. Zheng R. Xin J. Li S. Chu H. Gong W. (2018). Evaluation of GWAS-identified genetic variants for gastric cancer survival. *EBioMedicine* 33 82–87. 10.1016/j.ebiom.2018.06.028 29983348 PMC6085567

[B17] He Y. Wang C. Wang Z. Zhou Z. (2016). Genetic variant PLCE1 rs2274223 and gastric cancer: more to be explored. *Gut* 65 359–360. 10.1136/gutjnl-2015-309968 26078291

[B18] Huang da W. Sherman B. T. Lempicki R. A. (2009). Systematic and integrative analysis of large gene lists using DAVID bioinformatics resources. *Nat. Protoc.* 4 44–57. 10.1038/nprot.2008.211 19131956

[B19] Jukic I. Vukovic J. Rusic D. Bozic J. Bukic J. Leskur D. (2021). Adherence to maastricht V/florence consensus report for the management of *Helicobacter* pylori infection among primary care physicians and medical students in Croatia: a cross-sectional study. *Helicobacter* 26:e12775. 10.1111/hel.12775 33368852

[B20] Kang J. H. Lee S. I. Lim D. H. Park K. W. Oh S. Y. Kwon H. C. (2012). Salvage chemotherapy for pretreated gastric cancer: a randomized phase III trial comparing chemotherapy plus best supportive care with best supportive care alone. *J. Clin. Oncol.* 30 1513–1518. 10.1200/JCO.2011.39.4585 22412140

[B21] Li W. Y. Han Y. Xu H. M. Wang Z. N. Xu Y. Y. Song Y. X. (2019). Smoking status and subsequent gastric cancer risk in men compared with women: a meta-analysis of prospective observational studies. *BMC Cancer* 19:377. 10.1186/s12885-019-5601-9 31014273 PMC6480657

[B22] Li X. Li X. Jiang M. Tian W. Zhou B. (2018). Single nucleotide polymorphisms in PLCE1 for cancer risk of different types: a meta-analysis. *Front. Oncol.* 8:613. 10.3389/fonc.2018.00613 30619753 PMC6297376

[B23] Liang P. Zhang W. Wang W. Dai P. Wang Q. Yan W. (2019). PLCE1 polymorphisms and risk of esophageal and gastric cancer in a Northwestern Chinese population. *Biomed. Res. Int.* 2019:9765191. 10.1155/2019/9765191 30931333 PMC6413391

[B24] Lin X. J. Wang C. P. Liu X. D. Yan K. K. Li S. Bao H. H. (2014). Body mass index and risk of gastric cancer: a meta-analysis. *Jpn. J. Clin. Oncol.* 44 783–791. 10.1093/jjco/hyu082 24951830

[B25] Liu X. Zhang X. Wang Z. Chang J. Wu Z. Zhang Z. (2014). Genetic polymorphism of the phospholipase C epsilon 1 gene and risk of gastric cancer. *Chin. Med. J.* 127 2511–2517. 10.3760/cma.j.issn.0366-6999.20133123 24985593

[B26] Lunet N. Valbuena C. Vieira A. L. Lopes C. Lopes C. David L. (2007). Fruit and vegetable consumption and gastric cancer by location and histological type: case-control and meta-analysis. *Eur. J. Cancer Prev.* 16 312–327. 10.1097/01.cej.0000236255.95769.22 17554204

[B27] Lutz M. P. Zalcberg J. R. Ducreux M. Ajani J. A. Allum W. Aust D. (2012). Highlights of the EORTC St. gallen international expert consensus on the primary therapy of gastric, gastroesophageal and oesophageal cancer - differential treatment strategies for subtypes of early gastroesophageal cancer. *Eur. J. Cancer* 48 2941–2953. 10.1016/j.ejca.2012.07.029 22921186

[B28] Malik M. A. Srivastava P. Zargar S. A. Mittal B. (2014). Phospholipase C epsilon 1 (PLCE1) haplotypes are associated with increased risk of gastric cancer in Kashmir valley. *Saudi J. Gastroenterol.* 20 371–377. 10.4103/1319-3767.145330 25434319 PMC4271013

[B29] Mocellin S. Verdi D. Pooley K. A. Nitti D. (2015). Genetic variation and gastric cancer risk: a field synopsis and meta-analysis. *Gut* 64 1209–1219. 10.1136/gutjnl-2015-309168 25731870

[B30] Mou X. Li T. Wang J. Ali Z. Zhang Y. Chen Z. (2015). Genetic variation of BCL2 (rs2279115), neil2 (rs804270), LTA (rs909253), PSCA (rs2294008) and PLCE1 (rs3765524, rs10509670) genes and their correlation to gastric cancer risk based on universal tagged arrays and fe3o4 magnetic nanoparticles. *J. Biomed. Nanotechnol.* 11 2057–2066. 10.1166/jbn.2015.2113 26554163

[B31] Plummer M. Franceschi S. Vignat J. Forman D. de Martel C. (2015). Global burden of gastric cancer attributable to *Helicobacter* pylori. *Int. J. Cancer* 136 487–490. 10.1002/ijc.28999 24889903

[B32] Schwede T. Kopp J. Guex N. Peitsch M. C. (2003). SWISS-MODEL: an automated protein homology-modeling server. *Nucleic Acids Res.* 31 3381–3385. 10.1093/nar/gkg520 12824332 PMC168927

[B33] Shimizu T. Marusawa H. Matsumoto Y. Inuzuka T. Ikeda A. Fujii Y. (2014). Accumulation of somatic mutations in TP53 in gastric epithelium with *Helicobacter* pylori infection. *Gastroenterology* 147 407–417. 10.1053/j.gastro.2014.04.036 24786892

[B34] Smoot M. E. Ono K. Ruscheinski J. Wang P. L. Ideker T. (2011). Cytoscape 2.8: new features for data integration and network visualization. *Bioinformatics* 27 431–432. 10.1093/bioinformatics/btq675 21149340 PMC3031041

[B35] Song C. Hu C. D. Masago M. Kariyai K. Yamawaki-Kataoka Y. Shibatohge M. (2001). Regulation of a novel human phospholipase C, PLCepsilon, through membrane targeting by Ras. *J. Biol. Chem.* 276 2752–2757. 10.1074/jbc.M008324200 11022048

[B36] Szklarczyk D. Gable A. L. Lyon D. Junge A. Wyder S. Huerta-Cepas J. (2019). STRING v11: protein-protein association networks with increased coverage, supporting functional discovery in genome-wide experimental datasets. *Nucleic Acids Res.* 47 D607–D613. 10.1093/nar/gky1131 30476243 PMC6323986

[B37] Umar M. Upadhyay R. Mittal B. (2013). PLCE1 rs2274223 A>G polymorphism and cancer risk: a meta-analysis. *Tumour Biol.* 34 3537–3544. 10.1007/s13277-013-0932-7 23797815

[B38] Waterhouse A. Bertoni M. Bienert S. Studer G. Tauriello G. Gumienny R. (2018). SWISS-MODEL: homology modelling of protein structures and complexes. *Nucleic Acids Res.* 46 W296–W303. 10.1093/nar/gky427 29788355 PMC6030848

[B39] Xie Z. Wang B. Chai Y. Chen J. (2020). Estimation of associations between 10 common gene polymorphisms and gastric cancer: evidence from a meta-analysis. *J. Clin. Pathol.* 73 318–321. 10.1136/jclinpath-2019-206189 31767616

[B40] Xue W. Zhu M. Wang Y. He J. Zheng L. (2015). Association between PLCE1 rs2274223 A > G polymorphism and cancer risk: proof from a meta-analysis. *Sci. Rep.* 5:7986. 10.1038/srep07986 25614244 PMC4303865

[B41] Yu S. Choi W. I. Choi Y. J. Kim H. Y. Hildebrandt F. Gee H. Y. (2020). PLCE1 regulates the migration, proliferation, and differentiation of podocytes. *Exp. Mol. Med.* 52 594–603. 10.1038/s12276-020-0410-4 32238860 PMC7210307

[B42] Yuan S. Chan H. Filipek S. Vogel H. (2016). PyMOL and INKSCAPE bridge the data and the data visualization. *Structure* 24 2041–2042. 10.1016/j.str.2016.11.012 27926832

[B43] Zeng H. Chen W. Zheng R. Zhang S. Ji J. S. Zou X. (2018). Changing cancer survival in China during 2003-15: a pooled analysis of 17 population-based cancer registries. *Lancet Glob. Health* 6 e555–e567. 10.1016/S2214-109X(18)30127-X 29653628

