# Supplementary material for: PLCE1 Polymorphisms Are Associated With Gastric Cancer Risk: The Changes in Protein Spatial Structure May Play a Potential Role
Source: Front Genet. 2021 Aug 31;12:714915. doi: 10.3389/fgene.2021.714915 (PMC8438327; doi:10.3389/fgene.2021.714915)
Supplement: Supplementary file 1 [file Table_1.DOCX]

**TABLE S1 |** The allele/genotype and frequencies of SNPs of PLCE1 in gastric cancer patients and controls.

| **SNP ID** | **Allele** | **Case (%)** | **Control (%)** | **OR (95% CI)** | ***P* value** | **Genotype** | **Case (%)** | **Control (%)** | **OR (95% CI)** | ***P* value** |
| --- | --- | --- | --- | --- | --- | --- | --- | --- | --- | --- |
| rs3765524 | T | 287 (24.40) | 300 (21.34) | 1.19 (0.99-1.43) | 0.067 | TT | 29 (4.93) | 34 (4.84) | 1 | 0.080 |
|  | C | 889 (75.60) | 1104 (78.66) |  |  | TC | 229 (50.85) | 232 (33.00) | 0.86 (0.51-1.47) |  |
|  |  |  |  |  |  | CC | 330 (56.12) | 436 (62.02) | 1.13 (0.67-1.89) |  |
| rs2274223 | G | 289 (24.57) | 299 (21.27) | 1.20 (1.00-1.45) | **0.048^*^** | GG | 29 (4.93) | 33 (4.69% | 1 | 0.064 |
|  | A | 887 (75.43) | 1105 (78.73) |  |  | GA | 231 (39.29) | 233 (33.14) | 0.89 (0.52-1.51) |  |
|  |  |  |  |  |  | AA | 328 (55.78) | 436 (62.02) | 1.61 (0.95-2.72) |  |
| rs3781264 | C | 224 (19.05) | 199 (14.15) | 1.43 (1.16-1.76) | **0.001^*^** | CC | 17 (2.89) | 15 (2.13) | 1 | **0.002^*^** |
|  | T | 952 (80.95) | 1207 (85.85) |  |  | CT | 190 (32.31) | 169 (24.04) | 1.01 (0.49-2.08) |  |
|  |  |  |  |  |  | TT | 381 (64.80) | 519 (73.83) | 1.54 (0.76-3.13) |  |

Note: ORs and *P* values were adjusted by age and gender.

Abbreviations: OR: odds ratio; CI: confidence interval.

^*^: *P* <0.05 indicates statistical significance.
